# Targeting Protein-Tyrosine Phosphatases in Breast Cancer

**DOI:** 10.18632/oncotarget.496

**Published:** 2012-05-18

**Authors:** Nicola Aceto, Mohamed Bentires-Alj

**Affiliations:** Friedrich Miescher Institute for Biomedical Research, Basel, Switzerland; Friedrich Miescher Institute for Biomedical Research, Basel, Switzerland

Most signaling pathways are modulated by reversible tyrosine phosphorylation, which is regulated by the opposing actions of protein-tyrosine kinases (PTKs) and protein-tyrosine phosphatases (PTPs) [1]. Abnormal tyrosine phosphorylation underlies various diseases of deregulated growth and differentiation, including cancer [1]. Although the involvement of several PTKs in malignancy has been studied extensively (e.g., ErbB2/HER2 in breast cancer), elucidation of the participation of specific PTPs in this disease has only started recently. Here, we summarize the activities of two major PTPs, SHP2 [2] and PTP1B [3], in breast cancer, highlight their potential as targets for breast cancer treatment or prevention, and discuss the technical challenges facing the development of specific PTP inhibitors.

The tyrosine-phosphatase SHP2 has been shown to play a broad role in development and cancer, as well as in the regulation of cell fate and the activation of a number of signaling networks downstream of receptor tyrosine-kinases and cytokine receptors [4]. However, its merits as a drug target for breast cancer and its role in breast tumor progression have not been demonstrated previously. We have discovered a fundamental contribution of SHP2 to breast cancer progression and the propagation of tumor-initiating cells (TICs) *in vivo* [2]. Mechanistically, SHP2 activates transcription factors such as c-Myc and ZEB1 and induces the expression of a set of “SHP2 signature” genes found co-activated in a subset of human primary breast tumors and associated with invasive behavior and poor prognosis. SHP2, acting via c-Myc, induces the expression of the suppressor of miRNA biogenesis LIN28B which blocks the maturation of the tumor-suppressor microRNA let-7 [2]. These results provide new insights into signaling networks promoting tumor progression and TIC maintenance, as well as a rationale for developing SHP2-targeting agents for breast cancer therapy. In addition, the identification of a set of “SHP2 signature” genes, activated in the presence of SHP2 signaling, could provide a powerful tool to identify tumors eligible for SHP2-targeted therapy.

A further major PTP family member, PTP1B, is a well-established metabolic regulator that is associated with cancer [5]. For example, PTP1B knockout mice are insulin and leptin hypersensitive, which contributes to obesity-resistance under high-fat diet conditions [6, 7]. Moreover, whole-body deletion of PTP1B in mice delays or protects against HER2/Neu-induced mammary carcinogenesis [8, 9]. Recently, we found that deletion of PTP1B specifically in the mammary epithelium also delays the onset of HER2/Neu-evoked breast tumors, suggesting a cell-autonomous role for PTP1B in the onset of this disease [3]. However, deletion of PTP1B in established mouse mammary tumors or shRNA-mediated depletion of PTP1B in human breast cancer cell lines grown as xenografts did not affect tumor growth [3]. These results indicate that targeting PTP1B may be effective in breast cancer prevention but not in the treatment of advanced breast cancers of the HER2-positive subtype.

The latest findings on SHP2 and PTP1B clearly indicate that many patients would benefit from the design of specific inhibitors targeting these phosphatases. Unfortunately, the development of PTP inhibitors has encountered significant technical challenges. For example, interaction of inhibitor compounds with the PTP domain requires a high polarity, which is associated unfortunately with reduced cell permeability and bioavailability. To some extent, this problem can be addressed by the use of prodrugs or other chemical modifications. In addition, PTPs are highly susceptible to oxidation of the catalytic cysteine residue in the phosphatase domain, which leads to a conformational change at the active site and may limit binding efficacy. Taking advantage of this property, recent studies suggest that therapeutics that stabilize the oxidized form of the phosphatase and lock it in an inactive state may represent an alternative way for inhibiting PTPs [10]. Another important issue is the need to develop selective compounds that do not bind structurally similar PTPs, such as SHP1 (in the case of SHP2) or TC-PTP (in the case of PTP1B). This is a challenging aspect since PTPs share highly conserved phosphatase domains. However, specific PTP subpockets surrounding the active site can be targeted to enhance selectivity. Lastly, although these technical challenges may be overcome, there remains the challenge of identifying specific PTP substrates that can be used as biomarkers of the response to the inhibitor.

In conclusion, we are starting to uncover important activities of PTPs in breast cancer initiation, progression and maintenance. Studies of SHP2 and PTP1B have exposed them as potentially important targets for the treatment or prevention of breast cancer, not to mention the importance of PTP1B in diabetes. However, the appropriateness of specific inhibitors has to be fully confirmed, especially given the differential involvement of PTPs in an organ-dependent fashion. Crucial issues for future studies include the participation of other PTPs in tissue development and maintenance as well as cancer, and the signaling networks perturbed by PTP inactivation. This approach may lead to the discovery of novel signaling mechanisms regulated by PTPs and a better understanding of cancer-associated pathways.

## References

[1] Hunter T (2009). Current opinion in cell biology.

[2] Aceto N, Sausgruber N, Brinkhaus H (2012). Nature medicine.

[3] Balavenkatraman KK, Aceto N, Britschgi A (2011). Molecular cancer research : MCR.

[4] Chan G, Kalaitzidis D, Neel BG (2008). Cancer metastasis reviews.

[5] Yip SC, Saha S, Chernoff J (2010).

[6] Elchebly M, Payette P, Michaliszyn E (1999). Science.

[7] Klaman LD, Boss O, Peroni OD (2000). Molecular and cellular biology.

[8] Julien SG, Dube N, Read M (2007). Nature genetics.

[9] Bentires-Alj M, Neel BG (2007). Cancer research.

[10] Haque A, Andersen JN, Salmeen A (2011). Cell.

